# α/β-Hydrolase D16B Truncation Results in Premature Sperm Capacitation in Cattle

**DOI:** 10.3390/ijms23147777

**Published:** 2022-07-14

**Authors:** Shuwen Shan, Fangzheng Xu, Marc Hirschfeld, Claudia Herrmann, Martin Schulze, Ahmad Reza Sharifi, Michael Hoelker, Bertram Brenig

**Affiliations:** 1 Institute of Veterinary Medicine, University of Goettingen, 37077 Goettingen, Germany; shansw90@yahoo.com (S.S.); fangzheng.xu@nih.gov (F.X.); marc.hirschfeld@gmx.de (M.H.); 2 Malaria Functional Genomics Section, Laboratory of Malaria and Vector Research, National Institute of Allergy and Infectious Disease, National Institutes of Health, Bethesda, MD 20892, USA; 3 MVZ Brustzentrum Freiburg, 79110 Freiburg, Germany; 4 Institute for Reproduction of Farm Animals Schoenow, 16321 Bernau, Germany; c.herrmann@ifn-schoenow.de (C.H.); m.schulze@ifn-schoenow.de (M.S.); 5 Department of Animal Sciences, Devision Animal Breeding and Genetics, University of Goettingen, 37017 Goettingen, Germany; rsharif@gwdg.de; 6 Department of Animal Sciences, Division Biotechnology and Reproduction of Farm Animals, University of Goettingen, 37077 Goettingen, Germany; michael.hoelker@uni-goettingen.de

**Keywords:** premature capacitation, *ABHD16B*, loss of function, sperm membrane, lipid composition, male infertility, Holstein cattle

## Abstract

Recently it was shown that a specific form of male infertility in Holstein cattle was caused by a nonsense variant in the α/β-hydrolase domain-containing 16B (*ABHD16B*) gene resulting in a protein truncation at amino acid position 218 (p.218Q*) and loss of function. Lipidomics showed that the absence of ABHD16B influenced the content of phosphatidylcholine (PC), ceramide (Cer), diacylglycerol (DAG), and sphingomyelin (SM) in variant carrier sperm membranes. However, the exact cause of infertility in affected sires has remained unclear until now. To elucidate the cause of infertility, we analyzed (i) standard sperm parameters (i.e., total sperm number, morphological intact sperm, total sperm motility), (ii) in vitro fertilizability and effects on early embryonic development, and (iii) sperm survival rates (i.e., capacitation time). The affected spermatozoa showed no changes in the usual sperm parameters and were also capable of fertilization in vitro. Furthermore, the absence of ABHD16B did not affect early embryonic development. Based on these results, it was concluded that the affected spermatozoa appeared to be fertilizable per se. Consequently, the actual cause of the inability to fertilize could only be due to a time- and/or place-dependent process after artificial insemination and before fertilization. A process fundamental to the ability to fertilize after insemination is capacitation. Capacitation is a biochemical maturation process that spermatozoa undergo in the female genital tract and is inevitable for the successful fertilization of the oocyte. It is known that the presence and concentration of certain sperm membrane lipids are essential for the correct course of capacitation. However, precisely these lipids are absent in the membrane of spermatozoa affected by the ABHD16B truncation. Since all other causes of fertilization inability were excluded in the previous experiments, consequently, the only remaining hypothesis was that the loss of function of ABHD16B leads to a capacitation disruption. We were able to show that heterozygous and homozygous affected spermatozoa exhibit premature capacitation and therefore decay before fertilization. This effect of the loss of function of ABHD16B has not been described before and our studies now revealed why sires harboring the variant in the ABHD16B gene are infertile.

## 1. Introduction

The process of capacitation was initially described in the early 1950s [1,2]. During this process, spermatozoa compartments experience a series of physiological changes including the acquisition, loss, or redistribution of factors originating from the epididymis or seminal plasma [3]. These changes include, among others, reorganization of membrane proteins and lipids, substantial efflux of cholesterol together with an influx of bicarbonate ion (HCO_3_^−^), and initiation of calcium fluxes [4,5,6,7]. Despite some remaining controversy, capacitation mainly includes hyperactivation [8] and the acrosome reaction (AR) [9]. Hyperactivation of sperm allows them to fertilize an oocyte, including helping sperm escape from the pockets formed by mucosal folds [10], detaching sperm from adherence to the oviductal epithelium [11], facilitating sperm penetration through the viscous and viscoelastic substances such as oviductal mucus and the cumulus matrix [12,13], and promoting sperm penetration into zona pellucida [14,15]. Sperm membrane alteration is the crucial process during acrosomal reaction, thus outreaching the release of the acrosomal content intrinsically. The acrosome reaction is responsible for enabling the sperm to penetrate and fuse the oocyte [16].

In vivo, spermatozoa have to migrate long distances in the female from the vagina (site of deposition) to the upper part of the oviduct (site of fertilization) [9]. Fine control and regulation of capacitation are critical for spermatozoa to ensure that they reach the oocyte at the right time and in the right state [17]. Defects in hyperactivation may result in the inability of spermatozoa to be released from the oviductal reservoir and to penetrate the zona pellucida (ZP) [18]. On the other hand, early capacitation might deprive the spermatozoa of the ability to interact with oocytes successfully [9]. In humans, a high level of prematurely hyperactivated spermatozoa in semen was considered as a possible cause of idiopathic infertility [19]. Many studies have reported that cryopreservation of sperm will result in premature capacitation and it is related to lower fertilizing ability in bovines [20,21]. Premature capacitation can also occur if spermatozoa are exposed to toxic substances such as lead [22] and bisphenol A (BPA) [23]. Semenogelin (Sg) is the major human semen coagulation protein. Sg and its degradation products may regulate sperm capacitation and prevent this process from occurring prematurely [24]. NYD-SP27, an isoform of phospholipase C Zeta 1 (PLCZ1), has been reported to be a physiological inhibitor of PLC that prevents premature sperm capacitation and AR [25].

In a previous study, we detected a nonsense variant in the α/β-hydrolase domain-containing 16B (*ABHD16B*) gene (NC_037340.1g.53957903 G>A; ARS-UCD1.2; rs468948776) which was significantly associated with bull infertility in Holstein cattle [26]. Currently, we have very limited knowledge about the biochemical or physiological functions of ABHD16B. ABHD16B is known to belong to a large enzyme superfamily harboring an α/β-hydrolase structural domain [27]. ABHD proteins play an important role in lipid metabolism, lipid signaling and metabolic diseases. In human, ABHD2 participates in sperm hyperactivation as a lipid hydrolase by depleting endocannabinoid 2-arachidonoylglycerol (2-AG), an inhibitor of sperm calcium channel (CatSper) [28]. In previous studies, we detected the expression of ABHD16B in the nucleoplasm of bovine Leydig cells, in the seminiferous tubules, and in the epithelium of the epididymal duct at different intensities. These findings suggest that ABHD16B may play a role in spermatogenesis and sperm maturation in cattle. Due to the loss of function of ABHD16B an alteration of the spermatozoa membrane composition was observed. This included lower levels of diacylglycerol (DAG) and sphingomyelin (SM) and higher levels of phosphatidylcholine (PC) and ceramide (Cer). Since these lipids have been shown to play a major role in capacitation, we hypothesized that the variant-induced altered sperm membrane composition is causative for infertility [29,30,31,32].

## 2. Results

### 2.1. Spermatological Parameters

To examine the cause of infertility, the three most likely effects of the altered lipid composition of the sperm membrane were investigated. The first and initially most obvious changes could involve common spermatological parameters. Although such parameters are usually investigated before a sire is used in artificial insemination and there were no indications of this available for the homozygous carriers, it seemed reasonable to carry out a detailed investigation of these parameters first.

Standard spermatological parameters, i.e., general morphology (head, mid-piece, tail), thermoresistance test, total sperm motility, and sperm concentration were evaluated. None of these parameters showed significant differences between the three genotypes (Table 1).

**Figure 1:**
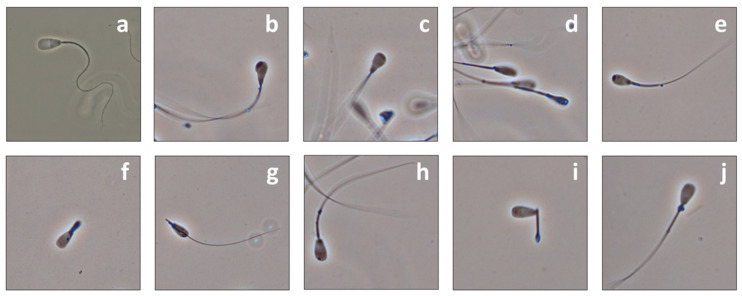
Morphology abnormalities of spermatozoa from homozygous carriers. In contrast to control individual (**a**) sperms of homozygous carriers showed head (**b**–**e**) and midpiece (**f**–**j**) anomalies. (**b**) Thickened apical region; (**c**) narrow postacrosomal region; (**d**) narrow sperm head size; (**e**) irregular heads with dense apical regions. (**f**) Swollen; (**g**) broken; (**h**) thickened midpiece; (**i**) cytoplasmic droplet (distal); (**j**) cytoplasmic droplet (proximal).

Although some morphological abnormalities of the sperm head and mid-piece were observed in the homozygous carriers, these were in the expected range of cryopreserved semen and would not explain infertility (Figure 1). Based on the results of the spermatological examinations, it was understandable that the semen samples of the affected bulls were released for artificial insemination without any concerns.

### 2.2. In Vitro Fertilization

**Figure 2:**
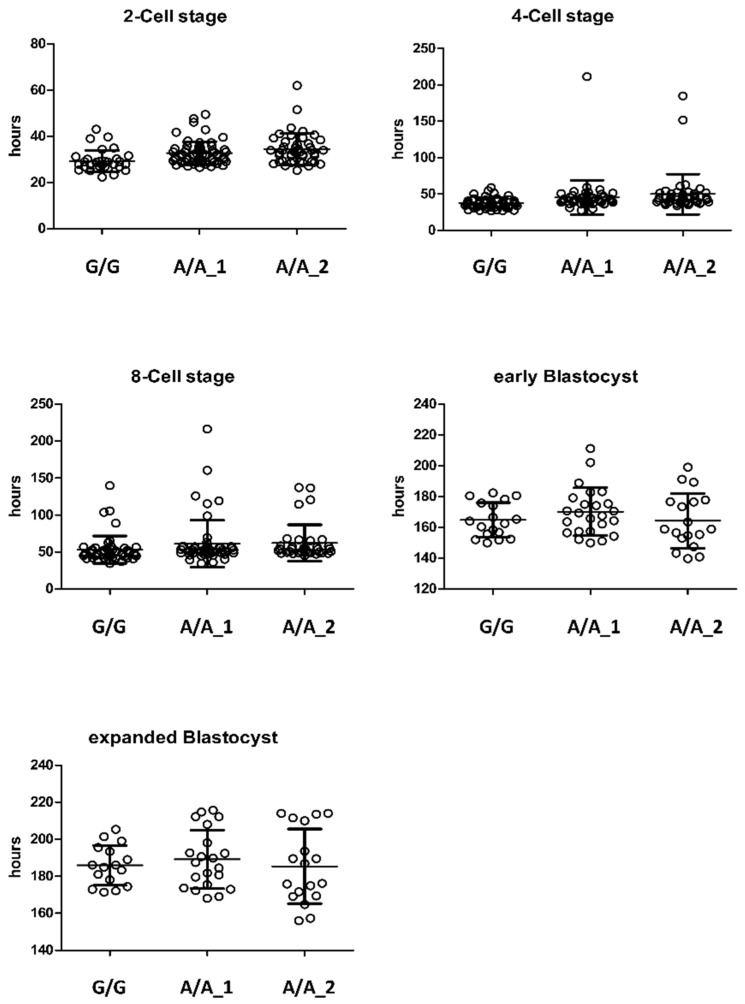
Effect of bull genotype on early embryo development. Data are presented with hours for early embryos to reach distinct stages after fertilizing in vitro generated with sperms from one wild-type and two homozygous carriers.

In the second approach, it was therefore tested whether the semen would fertilize in vitro. To investigate this, in vitro fertilization capacity of semen of two homozygous carriers (A/A_1, A/A_2) was compared to control samples (G/G). The early embryo development potential was followed until the expanded blastocyst stage as shown in Table 2 and Table 3. Cleavage rates of the early embryos (2-, 4-, 8-cell stage) generated with one homozygous carrier sample did not show significant differences compared to the wild-type control (84.2 ± 9.7% vs. 88.6 ± 4.3%). When using semen samples of a second homozygous carrier there was significant retardation observed in the development (62.6 ± 12.2%). In later stages of the development (hatched blastocyst), however, these differences disappeared (Figure 2). The observed difference in the developmental capacity of the second sire was therefore attributed to an individual effect rather than to the homozygous carrier genotype.

### 2.3. Sperm Capacitation Analysis

**Figure 3:**
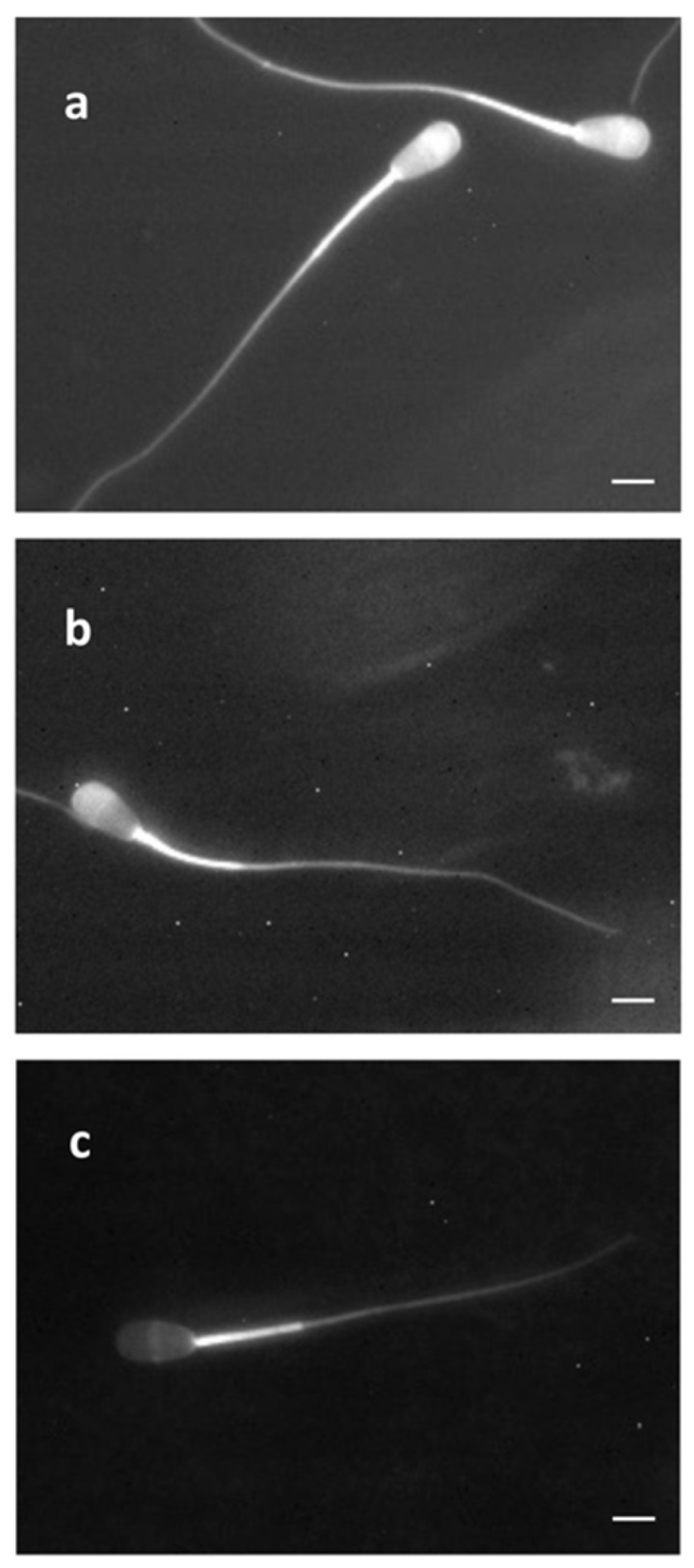
Chlortetracycline (CTC) fluorescence staining patterns of spermatozoa capacitation. (**a**) Uncapacitated, acrosome-intact sperm, with uniform fluorescence throughout the head (corresponds to group F in Table 4). (**b**) Capacitated, acrosome-intact sperm, with uniform fluorescence at the anterior head (corresponds to group B in Table 4). (**c**) Capacitated, acrosome-reacted spermatozoa, with a thin band only in the equatorial segment and faint fluorescence in the whole head (corresponds to group AR in Table 4). All capacitation states exhibit bright fluorescence in the midpiece. Bar = 5 µm.

Both experiments (spermatological properties, in vitro fertilizability) did not indicate any effects of the *ABHD16B* variant so far. Consequently, the only remaining possibility was that the affected spermatozoa lose their fertilizing ability after artificial insemination on their way through the female genital tract. Capacitation, the most important event in migration, was analyzed in semen of different *ABHD16B* genotypes. Freshly thawed samples were incubated and changes regarding capacitation and acrosome response were observed over a period of 2 h. The chlortetracycline (CTC) fluorescence was used to indicate capacitation status by assessing Ca^2+^-related changes [33]. Figure 3 shows the different states of the spermatozoa as used for assessment. The different states can be clearly differentiated microscopically.

**Figure 4:**
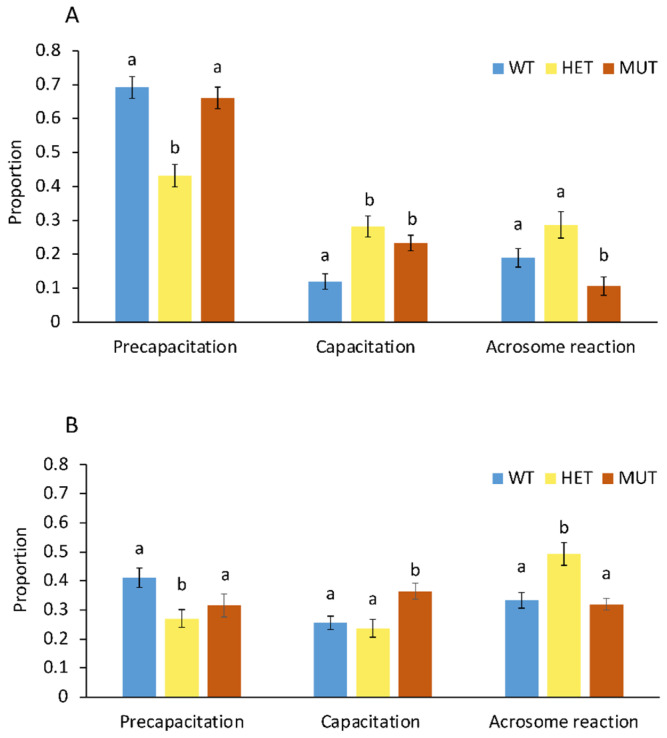
Effect of *ABHD16B* genotype on capacitation of spermatozoa**.** Three genotypes are shown as wild type (WT, blue bars), heterozygous (HET, yellow bars), and homozygous carriers (MUT, red bars). (**A**) Analysis of spermatozoa at the beginning of the incubation (0 h). (**B**) Analysis of spermatozoa after 2 h of incubation. Staining and evaluation of spermatozoa was carried out as described in the Materials and Methods section and also shown in Figure 3. Error bars indicate standard deviations. Small letters (a, b) show significant differences at a significance level of *p* < 0.05.

Numbers of spermatozoa at the different states were counted and statistically evaluated (Figure 4). In Figure 4A, spermatozoa are shown at the beginning of the incubation period (0 h). It can be seen that heterozygous carriers have a significantly lower percentage of spermatozoa (43.2%) before capacitation. The proportion of already capacitated spermatozoa was significantly increased in both heterozygous (28.2%) and homozygous (23.3%) carriers compared with wild type (11.9%). In contrast, spermatozoa that had not yet shown an acrosome reaction were significantly reduced in homozygous carriers at the beginning of the incubation period (10.6%). After the 2 h incubation (Figure 4B), the proportion of non-capacitated spermatozoa was significantly reduced in all three genotypes. Again, the heterozygous genotype was significantly reduced compared with the other genotypes. When comparing the capacitated spermatozoa, a significant increase was seen in the homozygous carriers (36.4%). However, differences of the proportions of non-capacitated and capacitated spermatozoa were less pronounced and even reduced in the wild-type and heterozygous genotypes. The proportions of acrosome-reacted spermatozoa were increased in all three genotypes. The significant difference in acrosome-reacted spermatozoa in the heterozygous genotype reflected the already increased proportion at the beginning of incubation and was therefore not surprising.

The results showed that frozen sperm of heterozygous and homozygous carriers have a significantly higher proportion of capacitated spermatozoa already after thawing a semen sample. In addition, spermatozoa of homozygous carriers capacitate significantly earlier.

## 3. Discussion

In bovines, analysis of semen quality (semen volume, sperm concentration, motility, and morphology) before artificial insemination is a routine process at artificial insemination (AI) stations. A predominant proportion of male fertility problems is related to sperm quality, such as quantity, morphology, or motility [34]. However, these primary tests cannot fully determine potential fertility levels of sires. Fertilization is a complex process involving multiple parameters, such as sperm potentials for migration, capacitation, acrosome reaction, ZP binding and penetration, and sperm-oocyte fusion [35]. As a consequence, bulls with normal spermatological parameters were used in routine insemination, but they are ultimately identified as sterile or infertile after hundreds or thousands of inseminations fail to conceive cows [35,36]. Another part of male infertility is idiopathic infertility caused mainly by genetic factors, which account for about 30–40% of cases [37]. In our study, none of the standard spermatological parameters were significantly different between the three analyzed *ABHD16B* genotypes. This suggested that infertility in *ABHD16B* mutant bulls was not due to reduced sperm quality, at least not on the in vitro level.

We, therefore, tested the homozygous *ABHD16B* carriers for their in vitro fertilization potential. Surprisingly, ABHD16B-deficient spermatozoa were able to fertilize oocytes in vitro. Even though embryos obtained from one of the homozygous carriers showed an early developmental delay, this difference disappeared at later stages. Hence, this effect was therefore attributed to an individual difference rather than the *ABHD16B* variant.

Conflicting in vitro and in vivo results suggested that detrimental effects may occur during the process of sperm migration through the female reproductive tract. It has been shown in knockout experiments in mice that migration-related genes are important for fertility. For example, PMIS2^−/−^ mice are infertile despite normal spermatological parameters because the affected spermatozoa cannot cross the uterotubal junction. In addition, binding to the zona pellucida is impaired. However, these cells can fertilize cumulus-surrounded oocytes in IVF [38]. Knockout of the proximal caputepididymal-specific ribonuclease 10 (Rnase10) in mice showed reproductive failure due to an impaired sperm-ZP binding and crossing of the uterotubal junction in the female reproductive tract [39]. Nevertheless, Rnase10^−/−^ spermatozoa were able to fertilize oocytes and exhibited accelerated capacitation, resulting in better overall IVF capacity than wild-type sperm [39].

A process essential for fertilization during migration through the female genital tract is known as capacitation. Only through capacitation do spermatozoa acquire the competence to fertilize an oocyte. On the other hand, premature capacitation has a potential negative impact on sperm reproductive fitness. *ABHD16B* is involved in lipid metabolism and its absence results in an alteration of lipid composition in the spermatozoa cell membrane that is associated with capacitation [26]. The different membrane composition resulted in a premature capacitation and acrosome reaction as demonstrated by the CTC assay. Changes in the distribution and composition of plasma membrane lipids are an important feature of sperm capacitation [3]. During capacitation, the synthesis of PC from phosphatidylethanolamine (PE) is increased [40] and levels of phosphatidylinositol (PI) and lysophosphatidylcholine (lyso-PC) are also increased in vivo in porcine sperm [41]. This is in agreement with our previous results that the ratios of PC and lyso-PC were elevated in heterozygous sperm [26]. Abnormal distribution of lipids probably causes sperm to partially mature when preparing semen samples before freezing. Meanwhile, sphingosine is hydrolyzed to ceramides during capacitation [29]. Sphingomyelinase-treated sperm showed a faster capacitation response and more spontaneous acrosome exocytosis compared with control sperm [42]. DAG can be hydrolyzed by diacylglycerol lipase to 2-arachidonoylglycerol to prevent sperm hyperactivity [28]. Therefore, a decrease in DAG may also lead to premature capacitation. Spermatozoa that underwent acrosome reaction were not significantly elevated in the three genotypes at 0 h. This is in agreement with the IVF results, where the ability of these sperm to fertilize in vitro was not different. However, this condition changed significantly after the 2 h incubation, with a significant increase in the proportion of capacitated and acrosome-reacted spermatozoa of the heterozygous and homozygous carriers compared with wild-type. In our previous study, we showed that heterozygous spermatozoa contained higher amounts of lyso-PC and Cer. Due to the fusogenic properties of lysophospholipids, the elevated amount could prepare spermatozoa for the acrosome reaction [3]. Sphingomyelin is heavily hydrolyzed into Cer during the normal capacitation and the AR process [29]. Both the addition of cell-permeable Cer and the increase in endogenous Cer can enhance the role of intracellular calcium as an effective inducer of cytokinesis. Ceramides trigger AR in capacitated sperm and enhance gamete response to progesterone [30]. Therefore, disruption of lipids in ABHD16B-deficient sperm membranes may be one of the reasons for early capacitation.

Hyperactivation is one of the important processes of sperm capacitation. It can be caused by in vitro calcium ionophores and progesterone released from nearby cumulus cells [28]. ABHD2 binds progesterone and acts as a progesterone-dependent lipid hydrolase, depleting endogenous 2-AG from the plasma membrane. The removal of 2-AG leads to the influx of calcium via CatSper and thus promotes sperm hyperactivation. ABHD16B has a similar hydrolase domain and may also be involved in calcium transport. Therefore, it is crucial to assess intracellular calcium levels in sperm incubated in culture medium at different time points. The limited availability of samples from homozygous carriers must be taken into account at this stage when assessing the results. However, due to the very low allele frequency of the variant in the Holstein population, it will be difficult to find further naturally occurring homozygous carriers in the future [26]. In addition, breeding programs strictly select against infertility. Additional supporting studies on hyperactivation, sperm calcium concentration, and protein tyrosine phosphorylation will therefore probably only be possible if homozygous carriers are bred experimentally in animal studies [43,44]. In conclusion, the altered lipid composition of the sperm membrane caused by the loss of function of ABHD16B most likely leads to premature capacitation and acrosome reaction. As a result, the affected sperm either do not reach the fertilizable oocyte at all or do not reach it in time for successful fertilization. Apart from the fact that ABHD16B belongs to a large family of enzymes mainly active in lipid metabolism, signaling, and regulation, nothing was previously known about its specific function. The present study is the first to assign a specific function to ABHD16B and its role in fertility.

## 4. Materials and Methods

### 4.1. Ethical Statement

The collection of samples was approved by the Lower Saxony State Office for Consumer Protection and Food Safety (33.19-42502-05-17A196) according to §8a Abs. 1 Nr. 2 of the German Animal Protection Law.

### 4.2. Animals and Samples

Holstein ovaries were obtained from a local slaughterhouse. All spermatozoa were obtained from deep-frozen semen collected at AI stations. The collection of samples was approved by the Lower Saxony State Office for Consumer Protection and Food Safety (33.19-42502-05-17A196), according to §8a Abs. 1 Nr. 2 of the German Animal Protection Law.

The specimens used in this study were selected based on their *ABHD16B* genotypes. The wild type (G/G) was defined according to the variant in *ABHD16B* (rs468948776) with standard fertility (NR*_dev_* > 2). In contrast, heterozygotes (G/A) had reduced fertility (−2 ≤ NR*_dev_* ≤ 2), while homozygotes carriers (A/A) were infertile (NR*_dev_* < −2) [26]. As homozygous carriers, we used the same bulls as described in ref. [26] with NR*_dev_* < −25.

### 4.3. Analysis of Spermatological Parameters

Deep-frozen semen from three wild-type (G/G), five heterozygous (G/A), and two homozygous affected (A/A) bulls were chosen to assess spermatological parameters. NucleoCounter SP-100^TM^ was used for the evaluation of sperm concentration as specified by Revision 1.5 of the User Guide Manual No. 991-0100 (ChemoMetec A/S, Lillerød, Denmark).

Total sperm motility (%) were recorded at 5, 30, 60, 120, 180, and 240 min post-thawing during a thermo-resistance test (TRT). Sperm motility was determined using the computer-assisted sperm analysis (CASA) system AndroVision^®^ (Version 1.2, Minitüb GmbH, Tiefenbach, Germany) equipped with a phase contrast microscope (AXIO Scope A1, Carl Zeiss Microscopy, Jena, Germany), a high-resolution camera (Basler avA1000-100gc, Basler, Germany), a TV adapter (60-C 1″ 1.0×, Zeiss, Jena, Germany), and an automated microscope warming stage (38 °C). Aliquots of 4–6 µL were filled onto a 38 °C pre-warmed slide (Menzel-Gläser, Thermo Scientific, Schwerte, Germany), covered with a regular cover slide (18 × 18 mm, Labsolute^®^, Th. Geyer GmbH, Berlin, Germany) and analyzed promptly (approximately 15–30 s). A minimum of 1000 sperm/sample was recorded in four to five frames (200× magnification) at a rate of 30 pictures per 0.5 s. According to the manufacturer’s default setting for bull semen, spermatozoa were defined as motile when the head activity (HAC) was ≥0.087 rad.

For the assessment of sperm morphology immediately after thawing, spermatozoa were fixed with 1% formalin in a phosphate-buffered solution (1 + 5). Two hundred spermatozoa per sample were evaluated using phase contrast microscopy (800× total magnification, Jenaval, Carl Zeiss, Jena, Germany). The percentages of morphologically normal spermatozoa and spermatozoa with abnormalities were evaluated according to [45].

Analyses of plasma membrane and acrosome integrity (PMAI) were performed using a Cytoflex S flow cytometer (Beckmann Coulter, Krefeld, Germany) equipped with a 488 nm solid-state laser. The sperm population was gated according to the expected forward- and sideward-scatter signals. A total of 10,000 events fitting the respective gate were counted. For incubation at 38 °C, a dry block heater was used (Techne Dri-Block^®^ DB2.D, Techne AG, Burkhardtsdorf, Germany). A double-staining flow cytometric method using propidium iodide (PI) and fluorescein-isothiocyanate conjugated peanut agglutinin (FITC-PNA) was used to differentiate between viable and dead spermatozoa and to characterize membrane integrity in the acrosomal region. Stock solutions (1 mg/mL) of FITC-PNA were diluted 1:10 with phosphate-buffered NaCl solution (26.8 mM Na_2_HPO_4_, 14.8 mM NaH_2_PO_4_, 102.6 mM NaCl; 305 ± 5 mOsmol/L; pH 7.0 at 20 °C), respectively. Aliquots of 375 µL diluted semen were mixed with 125 µL pre-warmed 0.5% PBS-buffered formalin and 12.5 µL FITC-PNA. After a 10 min incubation, 5 µL of 1.5 mM PI-solution was added. After incubation for 5 min at 38 °C in closed, lightproof tubes, aliquots of a 10 µL stained-sperm suspension were resuspended in a 2 mL pre-warmed PBS solution. Fluorescence signals of FITC-PNA, gathered via 533/30 nm band-pass filter, and PI, gathered via 670 nm long-pass filter, were plotted on logarithmic scales. The percentage of viable sperm with intact plasma and acrosomal membranes (PI-negative, PNA-negative) was determined as described previously [46].

### 4.4. Analysis of In Vitro Fertilization Capacity and Embryo Development

#### 4.4.1. In Vitro Maturation and Fertilization of Bovine Embryos

Holstein ovaries were obtained from a local slaughterhouse and brought to the lab in 30 °C saline within 3 h. Cumulus oocyte complexes (COCs) were aspirated from small follicles (2 to 8 mm) and COCs with a homogenous, evenly granulated ooplasm, surrounded by at least 3 layers of compact cumulus cells, were transferred to modified Tissue Culture Medium 199 (TCM, Sigma, Taufkirchen, Germany) supplemented with 4.4 mM HEPES, 33.9 mM NaHCO_3_, 2 mM pyruvate, 2.9 mM calcium lactate, 55 µg/mL gentamycin and 12% (*v*/*v*) heat-inactivated estrus cow serum. After washing COCs three times, they were cultured in groups of 50 in 400 µL modified TCM supplemented with 10 µg/mL FSH (FSH-p, Sheering, Kenilworth, NJ, USA) at 39 °C in a humidified atmosphere with 5% (*v*/*v*) CO_2_ in the air. Fertilization was performed in a Fert-TALP medium supplemented with 20 µM penicillinamine, 10 µM hypotaurine, 2 µM epinephrine, 6 mg/mL fatty acid-free BSA, 50 µg/mL gentamycin, and 1 µg/mL heparin [47]. For IVF, the deep-frozen semen of three bulls of different genetic backgrounds (two homozygous carriers (A/A_1 and A/A_2), one wild type (G/G)) were used. The wild-type sire served as lab control due to its proven suitability and fertility for in vitro production of bovine embryos. The final concentration of sperm in fertilization droplets was adjusted to 2 × 10^6^ sperms/mL. Following 18 h of co-culture, the presumptive zygotes were washed three times and were transferred to in vitro culture.

#### 4.4.2. In Vitro Culture of Bovine Embryos and Analysis of Developmental Competence

Embryo culture was performed in groups of approx. 50 in humidified atmosphere with 5% (*v*/*v*) CO_2_ in air at 39 °C for up to 9 days in 400 μL of SOFaa medium supplemented with 0.4% fatty acid-free bovine serum albumin (BSA-*FFA*) overlaid with mineral oil [48]. Developmental competence of embryos was recorded 48 h after placing the embryos into culture by counting the proportion of cleaved embryos and on days 7 and days 8 by counting the number of embryos that had reached early-, expanded-, or hatched blastocyst stage.

#### 4.4.3. Individual In Vitro Culture of Bovine Embryos for Morphokinetic Analysis of Embryo Development

Embryo culture was performed individually by taking advantage of a Miri^®^ Time-Lapse Incubator (Esco Medical ApS, Ega, Denmark) using the Miri^®^ Time-Lapse culture coins allowing individual culture of 14 embryos within each of six culture chambers in 5% (*v*/*v*) CO_2_ in air at 39 °C for up to 9 days in drops of 20 μL of SOFaa medium [48] supplemented with 0.4% BSA-*FFA* and overlaid with mineral oil. Analysis of individual morphokinetic development was conducted to assess the time (h) after onset of fertilization to reach distinct developmental stages (2-Cell stage, 4-Cell stage, 8-Cell stage, early blastocyst stage, and expanded blastocyst stage) for each embryo by use of the MIRI^®^ TL software version 2.1 (Esco Medical Technologies Ltd., Kaunas, Lithuania).

#### 4.4.4. Statistical Analysis of IVF Experiments

Data were analyzed by using the GLM procedure of Statistical Analysis System (SAS) version 8.0 (SAS Institute Inc., Cary, NC, USA) software package. Developmental rates of embryos cultured in groups were determined on a per well basis (approx. 50 oocytes) and were compared for different bulls by ANOVA followed by multiple pair-wise comparisons using Tukey test. Finally, values for hours after fertilization needed to reach distinct developmental stages were tested using ANOVA followed by multiple pair-wise comparisons using Tukey test. In each case, differences of *p* ≤ 0.05 were considered to be significant.

### 4.5. Analysis of Sperm Capacitation

#### 4.5.1. Culture Media

The media used for the washing and incubation of spermatozoa were modified Tyrode’s HEPES-buffered medium (HEPES-TALP: 114 mM NaCl, 3.1 mM KCl, 2.0 mM NaHCO_3_, 0.3 mM NaH_2_PO_4_, 2.1 mM CaCI_2_, 0.4 mM MgCl_2_, 0.2 mM Pyruvate, 10.0 mM sodium lactate, 10.0 mM HEPES, 10.0 mM Gentamycin, 3.0 mg/mL bovine serum albumin and 10.0 μg/mI Heparin) [47]. The bovine serum albumin (BSA, fatty acid-free) was obtained from Sigma Chemical Co. The chlortetracycline fluorescence solution was prepared with 750 µM CTC (Sigma), 130 mM NaCl, 5 mM cysteine (Sigma), and 20 mM Tris-HCl; the pH was adjusted to 7.8 [33,49]. The solution needed to be stored away from light and at a low temperature until use. All solutions needed to be adjusted to room temperature at the time of use.

#### 4.5.2. Sperm Suspension Preparation

Cryopreserved semen specimens of two wild type (G/G), one heterozygous carrier (G/A), and two homozygous affected (A/A_2 and A/A_3) were prepared. Deep-frozen semen was thawed by plunging straws into a 37 °C water bath for 30 s. SpermFilter (Gynemed GmbH & Co. KG, Lensahn, Germany), a density gradients regent, was used to purify and isolate life and highly motile spermatozoa according to the product instructions. Sperm were then washed two times in 2 mL HEPES-TALP medium, centrifuging at room temperature with 350× *g* for 10 min, and removing the supernatant. Resuspension of sperm cells with HEPES-TALP, and spermatozoa were assessed and standardized to 10^6^ spermatozoa/mL before performing the capacitation test [49]. 2.5 mL of sperm resuspension was placed in a 6-well cell culture plate and incubation was performed at 39 °C (5% CO_2_ in air) for 2 h. We considered the sperm after 2 h of incubation to be alive because we screened these sperm with Spermfilter initially. Assessment of sperm motility after 2 h showed that about 70% of the sperm were motile. Meanwhile, loss of motility did not mean that the cells had died [49].

#### 4.5.3. Chlortetracycline Assessment

Detection of the capacitation status by chlortetracycline fluorescence assay as previously reported [21,49]. The capacitation of sperm that incubated for 0 h and 2 h were compared. We mixed 45 µL of sperm supernatant with 45 µL of CTC solution, followed by incubation at room temperature for 30 min. Finally, 15 µL of 4% formaldehyde were added to fix the samples for 15 min. Slides were performed by adding 10 µL of the mixed suspension on a clean slide, gently adding a coverslip, and sealed with colorless nail varnish. To avoid CTC fading, slides were kept in a dark wet chamber and evaluated immediately.

Sperm cells were assessed on a Zeiss microscope (Axioplan2 imaging, AxioCam MRm, Apotome) with blue-violet illumination (excitation at 400–440 nm and emission at 470 nm) for CTC patterns. Three different capacitation patterns were classified as shown in Figure 3 according to [49]. ‘F’ shows cells before capacitation characterized by uniform fluorescence throughout the head; ‘B’ indicates capacitated but acrosome-intact cells with a non-fluorescent band in the postacrosomal region, and ‘AR’ indicates the acrosome-reacted cells with the feature of rather faint head fluorescence, usually showing a thin band in the equatorial segment. Sperm cells were randomly selected to classify the CTC staining patterns. The total sperm count at 0 h and 2 h is shown in Table 4.

#### 4.5.4. Statistical Analysis of Capacitation Experiments

Statistical analysis of capacitation was performed using a linear logistic model. We performed statistical analysis by using the same genotypes as a group. First, the proportion of spermatozoa with different characterization to the total number of spermatozoa in the sample was determined. This proportion was categorized as a binary response variable of a binomial distribution (y*_i_*). The dependent variable (y*_i_*) can take the value 1 for the probability of the incident ($\pi_{i}$) or the value 0 for the non-occurrence of the incidence (1 − $\pi_{i}$). Data were then analyzed using the GLIMMIX procedure of SAS with the following generalized linear model

$$log\left[\frac{\pi_{i}}{1-\pi_{i}}\right]=\varphi +\alpha_{i}$$

where *π_i_* is the probability of occurrence of sperm with a specific characteristic, φ is the overall mean effect, $\alpha_{i}$ is the fixed effect of the genotype. Least squares means were estimated on the logit scale and then back-transformed using the inverse link function *π* = exp(*xß*)/[1 + exp(*xß*)] to the original scale. Significant differences between least squares means were tested using a *t*-test procedure by inclusion of the PDIFF option in the LSMEANS statement (SAS) and adjusted by Tukey–Kramer correction.

## Figures and Tables

**Table 1:** Spermatological parameters analysis of deep-frozen semen in bulls of three genotypes.

Spermatological Parameter		G/G	G/A	A/A
		(Mean ± SEM)	(Mean ± SEM)	(Mean ± SEM)
Sperm morphology (%)	N	60.5 ± 4.3	55.2 ± 6.3	52.3 ± 0.8
	K	3.5 ± 0.9	1.6 ± 0.6	7.3 ± 4.8
	KK	26.0 ± 4.8	31.1 ± 6.4	24.0 ± 5.5
	KKD	0.2 ± 0.2	0.3 ± 0.2	2.8 ± 2.3
	M	4.0 ± 1.3	4.3 ± 1.5	5.0 ± 1.5
	prP	1.2 ± 0.7	1.8 ± 1.1	3.0 ± 0.5
	distP	0.7 ± 0.3	2.6 ± 1.4	1.8 ± 0.3
	PS	1.7 ± 0.7	1.7 ± 0.4	0.5 ± 0.0
	S	2.3 ± 1.2	1.3 ± 0.3	3.3 ± 1.3
	MB	0.0 ± 0.0	0.1 ± 0.1	0.3 ± 0.3
Post-thaw total sperm motility after TRT	5 min	61.7 ± 4.4	69.0 ± 4.6	70.0 ± 5.0
	30 min	61.7 ± 4.4	69.0 ± 6.2	72.5 ± 2.5
	60 min	60.0 ± 2.9	67.0 ± 5.1	72.5 ± 2.5
	120 min	61.7 ± 3.3	60.0 ± 9.4	67.5 ± 2.5
	180 min	56.7 ± 1.7	49.0 ± 15.2	67.5 ± 2.5
	240 min	35.0 ± 10.4	34.0 ± 11.6	40.0 ± 5.0
Plasma membrane and acrosome intact spermatozoa (%)	0 min	49.2 ± 5.5	55.9 ± 5.0	55.7 ± 3.5
	180 min	41.4 ± 2.4	48.8 ± 5.2	44.1 ± 3.1
	240 min	35.4 ± 0.2	44.8 ± 5.4	38.0 ± 3.2
Sperm concentration (10^6^/mL)		86.4 ± 25.5	66.1 ± 10.4	67.8 ± 13.7

Ten straws of deep-frozen semen were used to examine the sperm parameters (*n* = 3/5/2 for wild type (G/G)/heterozygous (G/A)/homozygous carriers (A/A)). No significant differences in all parameters were detected among the three genotypes (*p* > 0.05). Acronyms of parameters: N = morphological intact spermatozoa; K = abnormal head; KK = secondary apical ridge defect; KKD = malformed head cap; M = different alterations of the mid-piece (par- or retro axial, malformed or folded, broken neck); proxP = proximal cytoplasmic droplet; distP = distal cytoplasmic droplet; PS = loop with internal plasma droplet; S = bent tail; MB = sperm with multiple deformities; TRT = thermo-resistance test.

**Table 2:** Effect of sire genotype on in vitro developmental rates (%) after in vitro fertilization.

				Development to Blastocysts		
	Replicates	Total	Cleaved	Day 7	Day 9	Day 9 Hatched
Bull Genotype	(*n*)	(*n*)	(Mean ± STD)	(Mean ± STD)	(Mean ± STD)	(Mean ± STD)
G/G	10	551	88.6 ± 4.3 ^a^	19.2 ± 7.6 ^a^	35.3 ± 5.7 ^a^	40.6 ± 22.1
A/A_1	10	535	84.2 ± 9.7 ^a^	19.0 ± 5.9 ^a^	33.8 ± 13.5 ^a^	40.0 ± 16.4
A/A_2	10	610	62.6 ± 12.2 ^b^	8.3 ± 10.5 ^b^	15.8 ± 13.0 ^b^	27.7 ± 24.6

G/G: Wild-type sample; A/A_1,2: Homozygous carrier samples; different superscripts indicate significant differences (*p* ≤ 0.05, ANOVA, Tukey test).

**Table 3:** Effect of sire genotype on hours (h) after fertilization to reach distinct morphological embryonic stages.

	Total	2-Cell Stage	4-Cell Stage	8-Cell Stage	Early Blastocyst	Expanded Blastocyst
Bull Genotype	(*n*)	(Mean ± STD)	(Mean ± STD)	(Mean ± STD)	(Mean ± STD)	(Mean ± STD)
G/G	70	29.3 ± 15.0 ^a^	37.7 ± 17.6 ^a^	53.2 ± 28.8	164.9 ± 72.8	185.9 ± 78.8
A/A_1	70	32.7 ± 12.4 ^b^	45.3 ± 28.0 ^ab^	61.2 ± 38.2	170.2 ± 81.8	189.1 ± 87.7
A/A_2	70	34.5 ± 17.6 ^b^	49.9 ± 32.6 ^b^	61.2 ± 38.2	164.2 ± 72.8	185.4 ± 82.2

G/G: Wild-type sample; A/A_1,2: Homozygous carrier samples; different superscripts indicate significant differences (*p* ≤ 0.05, ANOVA, Tukey Test).

**Table 4:** Sperm count for capacitation pattern characterization.

Genotype	0 h			2 h			Total
	F	B	AR	F	B	AR	
WT	139	24	38	90	56	73	420
HET	92	60	61	56	49	102	420
MUT	156	55	25	71	82	72	461
Total	387	139	124	217	187	247	

F: Sperm cells before capacitation; B: Capacitated sperm cells; AR: Acrosome reacted sperm cells; WT: Wild type; HET: Heterozygous carrier; MUT: Homozygous carrier.

## Data Availability

All available data are included in the manuscript. There are no additional materials.
